# Treatment satisfaction is associated with improved quality of life in patients treated with inhaled treprostinil for pulmonary arterial hypertension

**DOI:** 10.1186/1477-7525-11-31

**Published:** 2013-03-06

**Authors:** Hubert Chen, Erika B Rosenzweig, S Karl Gotzkowsky, Carl Arneson, Andrew C Nelsen, Robert C Bourge

**Affiliations:** 1 Department of Medicine, University of California San Francisco, 1 DNA Way, Mailstop 453A, South San Francisco, CA 94080, USA; 2 Genentech, Inc, South San Francisco, CA, USA; 3 Department of Pediatrics, Columbia University, New York, NY, USA; 4 United Therapeutics, Corp, Research Triangle Park, NC, USA; 5 University of Alabama at Birmingham, Birmingham, AL, USA

**Keywords:** Pulmonary arterial hypertension, Treatment satisfaction, Quality of life, Inhaled prostacyclin, Iloprost, Treprostinil

## Abstract

**Background:**

Patient treatment satisfaction is likely to be a highly relevant outcome measure in pulmonary arterial hypertension (PAH), a condition for which the benefits of treatment must be weighed against frequent, undesirable side effects, inconvenience, and complications associated with therapy. In this study, we sought to evaluate the psychometric properties of a patient-reported treatment satisfaction measure and its relationship to quality of life (QoL) among patients transitioning from inhaled iloprost (iILO) to inhaled treprostinil (iTRE).

**Methods:**

We studied treatment satisfaction among 66 subjects with PAH in a single-arm, open-label, multi-center trial of iTRE following transition from iILO. Treatment satisfaction was assessed using the Treatment Satisfaction Questionnaire for Medication (TSQM, version 1.4) administered to subjects immediately before and 12 weeks after transition of inhaled therapy. The TSQM is comprised of 4 domains: *effectiveness, side effects, convenience*, and *global satisfaction*. Scores range from 0 to 100 with higher scores indicating greater satisfaction. Six-minute walk distance (6MWD), functional class, adverse events, drug administration time, and PAH-specific QoL (CAMPHOR) were concurrently assessed.

**Results:**

Domains of the TSQM demonstrated evidence of strong internal consistency at baseline and at 12 weeks (Cronbach α = 0.88-0.93). Transition from iILO to iTRE was associated with an improvement in 3 of 4 TSQM domains: *effectiveness* (+20 ± 21, p < 0.0001), *side effects* (0 ± 22, p = 0.97), *convenience* (+39 ± 26, p < 0.0001), and *global satisfaction* (+20 ± 24, p = 0.0005). Change in *effectiveness* scores correlated with change in 6MWD (r = 0.43, p = 0.0004) and *side effects* scores at 12 weeks correlated inversely with number of severity-weighted treatment-emergent adverse events (r = −0.44, p = 0.0002). In multiple regression models adjusted for baseline characteristics, changes in *effectiveness* and *convenience* satisfaction scores were significantly associated with improvement in PAH-specific QoL (p = 0.002 and p = 0.01).

**Conclusions:**

The TSQM demonstrated acceptable performance characteristics in patients with PAH. Changes in treatment satisfaction resulting from transitioning from iILO to iTRE were associated with improvements in PAH-specific QoL.

## Introduction

Pulmonary arterial hypertension (PAH) is a life-threatening disorder of the pulmonary vasculature historically associated with poor survival [1]. Advances in the diagnosis and treatment of PAH over the past two decades appear to have modestly improved overall survival [2-4]. Despite improvements in life expectancy, quality of life (QoL) for those living with the condition remains significantly impaired [5-7]. While several medications have been shown to improve 6-minute walk distance (6MWD), the benefits of treatment may be offset by intolerable side effects, need for frequent monitoring, complicated delivery systems, and the time required to prepare and administer therapy. Indeed, qualitative studies designed to characterize PAH from the patient’s perspective confirm that accommodating treatment represents a substantial burden to patients and has a negative impact on QoL [8-10].

In recent years, inhaled prostacyclin analogues have emerged as an attractive treatment option [11]. Two inhaled prostacyclin analogues are currently approved in the United States for the treatment of PAH: iloprost and treprostinil [12,13]. Both have been demonstrated in clinical trials to increase exercise capacity in patients with World Health Organization (WHO) Group 1 PAH [14-16]. Due to a relatively short half life (20–30 minutes), iloprost has a recommended dosing regimen of 6 to 9 inhalation sessions per day, each lasting 4 to 10 minutes. In contrast, treprostinil, which has a substantially longer half-life (4.5 hours), can be administered 4 times per day, each session lasting 2–3 minutes, with a target maintenance dose of 9 breaths per session.

In a 12 week, multicenter, prospective, open-label trial of stable PAH patients transitioned from inhaled iloprost to inhaled treprostinil, total time spent on daily treatment activities was reduced by an average of 1.4 hours per day [17,18]. Significant improvement in 6MWD and QoL scores were also observed. In that trial, data on overall treatment satisfaction was concurrently assessed using a recently validated patient-reported outcome (PRO) measure, the Treatment Satisfaction Questionnaire for Medication (TSQM) [19]. The concept of treatment satisfaction—although still relatively novel—has gained increasing recognition over recent years [20-22]. Of the few instruments available to assess satisfaction with treatment, the TSQM is one of the most widely used. It has been applied across various conditions and different modalities of treatment [19,23-25]. For example, the validation cohort used to develop the TSQM included common conditions such as migraine, arthritis, hypertension, asthma, and diabetes, among others [19]. In that study, different routes of treatment administration were also evaluated, including oral, injection, topical, and inhalation. Despite the obvious importance of treatment satisfaction in conditions requiring intensive therapy, it has not yet been studied in PAH. Because the construct integrates the positive, as well as negative aspects of therapy, treatment satisfaction may be a particularly relevant outcome for patients with PAH.

Using data collected within the context of a clinical trial, we sought to evaluate the psychometric characteristics of the TSQM assessed among patients with PAH transitioning from inhaled iloprost to inhaled treprostinil. In addition, we sought to determine whether changes in treatment satisfaction were associated with observed changes in QoL.

## Methods

### Subjects

For this analysis, we utilized clinical and patient-reported outcome data obtained from a multicenter, prospective, open-label trial in patients with stable PAH rapidly transitioned from inhaled iloprost to inhaled treprostinil [17,18]. In brief, eligible patients were between the age of 18 and 75 years with a diagnosis of idiopathic/hereditary PAH, PAH associated with collagen vascular disease or human immunodeficiency virus, or PAH associated with congenital systemic-to-pulmonary shunt. Patients were required to have a baseline 6MWD of ≥250 meters(m) and be receiving a stable dose of iloprost for ≥30 days prior to baseline. Patients were considered ineligible if they had changed or discontinued any FDA-approved PAH medication within 30 days. Following completion of all baseline study assessments, patients discontinued iloprost therapy during the baseline visit and initiated inhaled treprostinil at 3 breaths (6 μg/breath) four times daily (qid) at their next scheduled dose of iloprost therapy. The suggested treprostinil dose titration was an increase of one additional breath per dosing session every three days with a goal of 9 breaths qid within the first 3 weeks of treatment. If clinically indicated, investigators were allowed to increase to a maximum dose of 12 breaths qid. Following institutional review board approval, all patients provided informed consent before any study-related assessments.

### Study assessments

Analysis was restricted to key outcomes of interest collected at baseline and week 12: 6MWD, QoL, treatment satisfaction, and medication administration time. In addition, we included a Patient Impression of Change scale administered at week 12 and data on study-emergent adverse events (AEs).

Treatment satisfaction was assessed using the TSQM (version 1.4) [19]. This questionnaire is comprised of 14 items which represent 4 domains: *effectiveness*, *side effects*, *convenience*, and *global satisfaction*. TSQM scores range from 0 to 100, higher scores indicating greater satisfaction. Quality of life was assessed using the Cambridge Pulmonary Hypertension Outcome Review (CAMPHOR) [26]. The CAMPHOR includes 3 separate subscales designed to assess symptoms, functioning, and QoL. The QoL subscale is comprised of 25 items defined using a “needs-based” conceptual model, which postulates that life gains its quality from the ability and capacity of the individual to satisfy his or her needs [27]. The CAMPHOR QoL subscale ranges from 0 to 25 with lower scores representing better QoL. Medication administration time was assessed using a drug administration activities survey for which patients were asked to provide information related to the frequency of daily administration activities and time required by each activity (e.g., gathering and setting up supplies, preparing drug delivery system, delivering inhalation treatment, and cleaning drug delivery system) for inhaled iloprost (baseline) and inhaled treprostinil (week 12). AEs were summarized using a severity-weighted AE score calculated as the sum of the 15 most common study-emergent AEs, where each event was scored as 0 if the event never occurred, 1 if the event was at most mild, 2 if the event was at most moderate, and 3 if the event was severe. Potential scores range from 0 (least burden) to 45 (most burden). To independently assess the magnitude of change in treatment satisfaction over time, we used a global Patient Impression of Change measure comprised of a single item with a 5-point Likert scale (“much more satisfied”, “more satisfied”, “about the same”, “less satisfied”, “much less satisfied”).

### Analysis

Sample size for the original prospective, open-label trial was based on anticipated enrollment over the planned timeline and not based on statistical power. As this trial was intended primarily to characterize the safety of the switch in therapies, the enrolled sample size of 73 was deemed adequate for this purpose. For the present analysis, only data for those subjects who completed the TSQM at baseline and week 12 were included. Of 73 subjects enrolled in the original trial, 7 were not included in this analysis due to missing TSQM data. The proportion non-idiopathic PAH was higher in the excluded subjects (71%) compared to those included in this analysis (50%), but they were otherwise similar in terms age, sex, race, WHO functional class, background PAH therapy, and 6-minute walk distance. Internal consistency was assessed using Cronbach’s alpha, a coefficient of reliability that indicates how closely related a set of items are to one another. Internal consistency was also assessed by examining intrascale correlations among the individual domains of the TSQM. Convergent validity of TSQM domains was assessed in relation to absolute change in 6MWD from baseline to week 12, percent change in total medication administration time from baseline to week 12, and severity-weighted AE score calculated for all AEs collected up to week 12. Based on the non-normal distribution exhibited by certain variables, a non-parametric approach was applied for all correlations (i.e., Spearman’s coefficients). Responsiveness of the TSQM and its individual domains was assessed in relation to the Patient Impression of Change scale. Longitudinal change in TSQM scores approximated a normal distribution, thus parametric testing for differences among Patient Impression of Change categories was performed using analysis of variance (ANOVA). The relationship between change in TSQM and change in CAMPHOR QoL scores was evaluated using simple (unadjusted) and multiple (adjusted) linear regression. Covariates in adjusted analyses included age, sex, race, etiology of PAH (idiopathic vs. non-idiopathic), WHO functional class, and type of background therapy (endothelin receptor antagonist and phosphodiesterase inhibitor). Statistical analyses were performed using SAS® software, version 9.2 (SAS Institute Inc., Cary, NC).

## Results

Baseline characteristics for 66 subjects included in this analysis are shown in Table 1. Mean age was 49 years, with a range 18–79 years. Subjects were predominantly female (77%) and white (88%). Half (50%) of subjects had idiopathic/heritable PAH. At baseline, 58% subjects were WHO functional class II and 42% were functional class III. Mean ± standard deviation (SD) 6MWD at baseline was 395 ± 79 m.

**Table 1:** Baseline characteristics of 66 subjects with PAH on inhaled prostacyclin

**Characteristic**	**Baseline (n = 66)**
Age, mean (range), yr	49 (18 – 74)
Male : Female, n (%)	15 (23) : 51 (77)
White*	58 (88)
PAH etiology, n (%)	
Idiopathic/heritable	33 (50)
Collagen vascular disease	13 (20)
Congenital heart disease	17 (26)
HIV	3 (5)
Background PAH therapy, n (%)	
Endothelin receptor antagonist	56 (85)
Phosphodiesterase-5 inhibitor	46 (70)
Both	39 (59)
None	3 (5)
WHO functional class, n (%)	
II	38 (58)
III	28 (42)
6-minute walk distance, mean ± SD, m	395 ± 79
Borg dypnea score, mean ± SD	3.4 ± 2.1
Iloprost dose, n (%)	
2.5 μg	4 (6)
5.0 μg	62 (94)
Iloprost frequency	
< 6 times per day	25 (38)
≥ 6 times per day	41 (62)

* n = 58 (8 missing).

All subscales of the TSQM showed high internal consistency, both on inhaled iloprost (baseline) and on inhaled treprostinil (week 12), with Cronbach alphas ranging from 0.88 to 0.93 (Table 2). On inhaled iloprost (baseline), the *global satisfaction* domain of the TSQM correlated more strongly (r = 0.79) with *effectiveness* than with either *side effects* or *convenience* (r = 0.23 and 0.42). On inhaled treprostinil (week 12), a similar pattern of correlations was observed, but with a much stronger correlation between *global satisfaction* and *convenience* (r = 0.73).

**Table 2:** Internal consistency and intrascale correlations for the TSQM at baseline and at 12 weeks

	**Cronbach α**	**Effectiveness**	**Side effects**	**Convenience**
At baseline (on iloprost)				
Effectiveness	0.89	1.00		
Side effects	0.93	0.21 (p = 0.10)	1.00	
Convenience*	0.91	0.26 (p = 0.03)	0.13 (p = 0.29)	1.00
Global satisfaction	0.93	0.79 (p < 0.0001)	0.23 (p = 0.06)	0.42 (0.0005)
At 12 weeks (on treprostinil)				
Effectiveness	0.90	1.00		
Side effects	0.88	0.54 (p < 0.0001)	1.00	
Convenience	0.91	0.51 (p < 0.0001)	0.44 (p = 0.0002)	1.00
Global satisfaction^†^	0.93	0.78 (p < 0.0001)	0.52 (p < 0.0001)	0.73 (p < 0.0001)

* 1 missing.

† 2 missing.

As shown in Table 3, transition from inhaled iloprost to inhaled treprostinil was associated with a significant improvement in all TSQM domains (*effectiveness*: +20 ± 21 points, *convenience*: +39 ± 26 points, *global satisfaction*: +20 ± 24 points), except for *side effects*, which remained approximately the same (0 ± 22 points). Of the 4 TSQM domains, convenience demonstrated the greatest increase. Parallel improvements were observed for 6MWD and medication administration time. From baseline to week 12, mean 6MWD improved by 16 ± 35 m and mean total medication administration time decreased by 80 ± 81 minutes/day. Reduction in the time required for inhalation (decrease of −53 ± 37 minutes/day) accounted for the largest proportion of the reduction in total medication administration time.

**Table 3:** Changes in TSQM, 6MWD, medication administration time, and adverse events associated with transitioning from inhaled iloprost to inhaled treprostinil

	**Baseline**	**Week 12**	**Absolute change**	**Standard effect size**	**p**
TSQM, mean ± SD					
Effectiveness	62 ± 10	82 ± 16	+20 ± 21	2.0	<0.0001
Side effects	85 ± 21	85 ± 18	0 ± 22	0	0.97
Convenience	44 ± 24	83 ± 17	+39 ± 26	1.6	<0.0001
Global satisfaction	61 ± 23	82 ± 18	+20 ± 24	0.9	<0.0001
6-minute walk distance, mean ± SD, m	395 ± 79	414 ± 84	+16 ± 35	0.2	0.0005
Medication administration time, mean ± SD, min./day					
Gather supplies	21 ± 24	12 ± 11	−9 ± 26	0.4	0.01
Prepare system	11 ± 8	9 ± 9	−2 ± 12	0.3	0.16
Inhalation	66 ± 35	13 ± 7	−53 ± 37	1.5	<0.0001
Clean	21 ± 47	5 ± 4	−16 ± 46	0.3	0.01
Total time	119 ± 78	39 ± 25	−80 ± 81	1.0	<0.0001
Adverse events, median (IQR)					
Severity-weighted adverse event score	-	4.4 ± 2.8	-	-	-

*IQR*: interquartile range.

6-minute walk distance missing for 2 subjects.

Medication administration time missing data for 5 subjects.

Convergent validity of the TSQM subscales was evaluated with respect to related study measures assessed concurrently (Table 4). Change in TSQM scores from baseline to week 12 correlated with change in 6MWD in the expected manner, with the *effectiveness* domain demonstrating the strongest correlation. Changes in TSQM scores did not correlate significantly with percent change in medication administration time. TSQM scores at week 12 correlated with severity-weighted AE score in the expected manner (inversely), with the *effectiveness* and *side effects* domains demonstrating the strongest correlations.

**Table 4:** Correlation between TSQM and 6MWD, medication administration time, and adverse events

	Change in 6-minute walk distance*	Percent change in medication administration time*	**Severity-weighted adverse event score**^**†**^
TSQM			
Effectiveness	0.43 (p = 0.0004)	0.04 (p = 0.79)	−0.45 (p = 0.0002)
Side effects	0.18 (p = 0.15)	0.18 (p = 0.17)	−0.44 (p = 0.0002)
Convenience	0.24 (p = 0.06)	0.14 (p = 0.28)	−0.36 (p = 0.003)
Global satisfaction	0.29 (p = 0.02)	0.14 (p = 0.30)	−0.35 (p = 0.005)

* Compared to change in TSQM from Baseline to Week 12.

† Compared to TSQM at Week 12.

Medication administration time missing data for 5 subjects.

Responsiveness of the TSQM to change was evaluated with respect to overall Patient Impression of Change (Table 5). Of 61 patients who completed the Patient Impression of Change item, 42 (69%) responded “much more satisfied”, 18 (30%) responded “more satisfied”, and 1 (2%) responded “less satisfied”. Subjects who were “much more satisfied” had greater improvement in *global satisfaction* (+23 ± 24 points), as measured by the TSQM, than subjects who were “more satisfied” (+14 ± 22 points) or “less satisfied” (−21 ± 0 points). A similar pattern was observed for the *effectiveness* and *convenience* domains, but not for *side effects*. Standardized effect sizes observed (Table 3) also supported the overall responsiveness of the instrument.

**Table 5:** Responsiveness of TSQM to patient-assessed global impression of change

	Patient impression of change (Baseline to week 12)*			
	Same or less satisfied* (n = 1)	**More satisfied (n = 18)**	**Much more satisfied (n = 42)**	**p**^**†**^
Change in TSQM, mean ± SD				
Effectiveness	−28 ± 0	+13 ± 18	+23 ± 21	0.02
Side effects	−75 ± 0	−2 ± 20	+3 ± 20	0.001
Convenience	+6 ± 0	+22 ± 18	+45 ± 26	0.002
Global satisfaction	−21 ± 0	+14 ± 22	+23 ± 24	0.10

Positive change in score indicates improvement.

5 missing.

* Includes any responses for “about the same”, “less satisfied”, or “much less satisfied”.

† ANOVA.

The relationships between change in TSQM scores and change in QoL, as measured by the CAMPHOR, are shown in Table 6. Observed changes in *effectiveness*, *convenience*, and *global satisfaction* were associated with change in QoL, with greater satisfaction corresponding to greater QoL. Change in *side effects* was not significantly associated with QoL. A similar pattern of findings was observed after adjusting for subject characteristics. Of the 4 domains, *effectiveness* explained the greatest proportion of variance in QoL scores (adjusted Model R^2^ = 0.12).

**Table 6:** Relationship between change in TSQM and change in CAMPHOR QoL score

**Predictor**	**Model R**^**2**^	**Standardized β ± SE**	**p**
Unadjusted Model			
Effectiveness	0.13	−0.07 ± 0.02	0.002
Side effects	<0.01	−0.01 ± 0.02	0.81
Convenience	0.09	−0.05 ± 0.02	0.008
Global satisfaction	0.06	−0.05 ± 0.02	0.03
Adjusted Model			
Effectiveness	0.12	−0.07 ± 0.02	0.002
Side effects	<0.01	−0.02 ± 0.03	0.50
Convenience	0.08	−0.05 ± 0.02	0.01
Global satisfaction	0.03	−0.04 ± 0.02	0.05

Adjusted model includes age, sex, race, etiology of PAH, WHO functional class, and type of background therapy.

## Discussion

Patients with PAH are confronted with a growing number of therapeutic options, each of which is associated with its own unique delivery system. Because little to no comparative effectiveness data exists, patient preferences can often be the driving factor behind choice of therapy. Thus, treatment satisfaction is likely to be highly relevant in the management of PAH. In this study, we evaluated the psychometric characteristics of a relatively new treatment satisfaction measure, the TSQM, assessed among patients participating in a 12 week open-label study of inhaled PAH therapy. We found that transitioning from inhaled iloprost to inhaled treprostinil was associated with significant improvements in treatment satisfaction as measured by the TSQM. Domains of treatment satisfaction impacted included *global satisfaction*, *effectiveness*, and *convenience*, but not *side effects*. Overall, the TSQM showed high internal consistency, good convergent validity, and evidence of responsiveness to change over 12 weeks. Increases in treatment satisfaction were significantly associated with improvement in QoL independent of subject characteristics.

Little is known about the determinants of treatment satisfaction in PAH. In a previous pilot study, we evaluated changes in treatment satisfaction using the TSQM among 10 patients with PAH transitioning from continuous intravenous epoprostenol to intravenous treprostinil [28]. In that study, a significant impact was observed in all domains of the TSQM at 8 weeks. Changes in treatment satisfaction were accompanied by improvement in QoL scores and a decrease in total time spent on drug preparation activities. Based on these favorable results, we utilized a similar approach in designing the current study of inhaled prostacyclin among a substantially larger population of patients with PAH [17,18]. Our findings support the reliability, validity, and responsiveness of the TSQM in PAH, while providing additional insights regarding its relationship with other health status measures.

In the case of inhaled prostacyclins, we found that *global satisfaction* with treatment appears to be most strongly associated with *effectiveness* of the medication as perceived by the patient. Significant correlations between change in treatment satisfaction and change in 6MWD further corroborate this finding. Nonetheless, *convenience* also appears to play a major role. Among the 4 treatment satisfaction domains, *convenience* appeared the most responsive to transition from inhaled iloprost to inhaled treprostinil. Parallel improvements in medication administration time were also observed, however, these changes did not correlate significantly with change in treatment satisfaction. One potential explanation for the lack of association is that additional factors (other than time spent administering medication) may have a significant impact on *convenience*. For example, particular features associated with the delivery device, such as ease of operation, need for cleaning/maintenance, and portability may be equally, or even more, important. Another explanation is that our study may not be adequately powered to detect weaker associations, although trends in the anticipated direction of effect were observed. In addition to *convenience*, *side effects* can also have a significant impact on treatment satisfaction as indicated by significant correlations with adverse events at week 12. In this study, however, we could not evaluate impact of changes in adverse events since they were not collected at baseline for iloprost. In spite of this, the lack of any change in the *side effects* domain of the TSQM suggests that treprostinil and iloprost may have similar side effect profiles.

Several limitations should be considered in the interpretation of our data. Most importantly, although the study was conducted in a prospective fashion, the relationships evaluated in our analysis utilized measures assessed concurrently. Consequently, causality cannot be inferred. Based on our findings, it is possible that improvement in 6MWD and reduction in medication administration time could have lead to the observed increase treatment satisfaction, but this remains speculative. Moreover, it is difficult to determine whether increased treatment satisfaction resulted in improved QoL, or vice versa. Cross-lagged panel models have been developed to address such temporal associations, but are require substantially more data. As previously mentioned, our small sample size also limits our ability to detect what may be modest, albeit meaningful relationships. Ideally, a varied range of responses is required in order to fully evaluate the responsiveness of an instrument. In the case of our results, our ability to demonstrate the responsiveness of the TSQM may have been limited by the fact that nearly all patients reported improvements in treatment satisfaction. Similar side effect profiles between the two inhaled prostacyclins may have also precluded our ability to assess responsiveness in this regard. Finally, our results are also vulnerable to potential selection bias insofar as patients who are currently dissatisfied with their current treatment (iloprost) may be more likely to have enrolled into the trial. Exclusion of patients with missing data for treatment satisfaction could have also introduced bias if the patients excluded were systematically different (e.g., more likely or less likely to be satisfied) than those included.

In summary, changes in treatment satisfaction in patients transitioning from inhaled iloprost to inhaled treprostinil were associated with increased exercise capacity and improved QoL. The TSQM demonstrates reasonable performance characteristics in patients with PAH and may be a useful tool for comparing satisfaction with PAH therapies, in particular, those requiring different delivery routes or dosing regimens.

## Competing interests

The authors declare that they have no competing interests.

## Authors’ contributions

HC contributed to the study hypothesis, wrote the data analysis plan, conducted the statistical analyses, interpreted the data, and drafted the manuscript. ER contributed to the study execution, participated in the interpretation of the data, and provided a critical review of the manuscript. KG contributed to the data analysis plan, participated in the interpretation of the data, and provided a critical review of the manuscript. CA contributed to the data analysis plan, participated in the interpretation of the data, and provided a critical review of the manuscript. AN contributed to the study hypothesis and data analysis plan, participated in the interpretation of the data, and provided a critical review of the manuscript. RB contributed to the study design and execution, participated in the interpretation of the data, and provided a critical review of the manuscript. All authors read and approved the final manuscript.

## Disclosures

HC has received consulting fees from United Therapeutics and research grant support from Pfizer. ER has received research grant support from Actelion and United Therapeutics, as well as honoraria for consultation on scientific advisory board for Actelion and United Therapeutics. KG, CA, and AN are employees of United Therapeutics and hold stock options with United Therapeutics. RB is a paid consultant, has received research grants from, and serves on the scientific advisory board of United Therapeutics. RB received stock options for United Therapeutics in 2009 for scientific advisory board services. RB has been a paid consultant and on advisory boards for Actelion. United Therapeutics has submitted data from this clinical study to the U.S. Patent Office to support additional claims on inhaled treprostinil. United Therapeutics financed this manuscript, including the article-processing charge.
